# Supplementary material for: USP36 stabilizes nucleolar Snail1 to promote ribosome biogenesis and cancer cell survival upon ribotoxic stress
Source: Nat Commun. 2023 Oct 13;14:6473. doi: 10.1038/s41467-023-42257-8 (PMC10575996; doi:10.1038/s41467-023-42257-8)
Supplement: Supplementary file 1 — Supplementary information [file 41467_2023_42257_MOESM1_ESM.pdf]

SUPPLEMENTARY INFORMATION

USP36 stabilizes nucleolar Snail1 to promote ribosome biogenesis and cancer cell survival upon ribotoxic stress

Kewei Qin[1*], Shuhan Yu[1*], Yang Liu[1], Rongtian Guo[1], Shiya Guo[1], Junjie Fei[1], Yuemeng Wang[1], Kaiyuan Jia[1], Zhiqiang Xu[2], Hu Chen[1,3], Fengtian Li[1], Mengmeng Niu[1], Mu-Shui Dai[4], Lunzhi Dai[2], Yang Cao[1], Yujun Zhang[1], Zhi-Xiong Jim Xiao[1,2#], Yong Yi[1#]

[1] Center of Growth, Metabolism and Aging, Key Laboratory of Bio-Resource and Eco-Environment of Ministry of Education, College of Life Sciences, Sichuan University, Chengdu, 610064, China.

[2] State Key Laboratory of Biotherapy, West China Hospital, Sichuan University, Chengdu, 610041, China.

[3] Department of Cardiothoracic Surgery, First Affiliated Hospital of Chengdu Medical College, Chengdu, 610500, China.

[4] Department of Molecular & Medical Genetics, Oregon Health & Science University, Portland, OR, USA.

[*]These authors contributed equally to this work

[#]Correspondence: yy-yiyong@scu.edu.cn (Yong Yi); jimzx@scu.edu.cn (Zhi-Xiong Jim Xiao)

Supplementary Figures S1-S10

Supplementary Table 1-4

Supplementary Figure S1

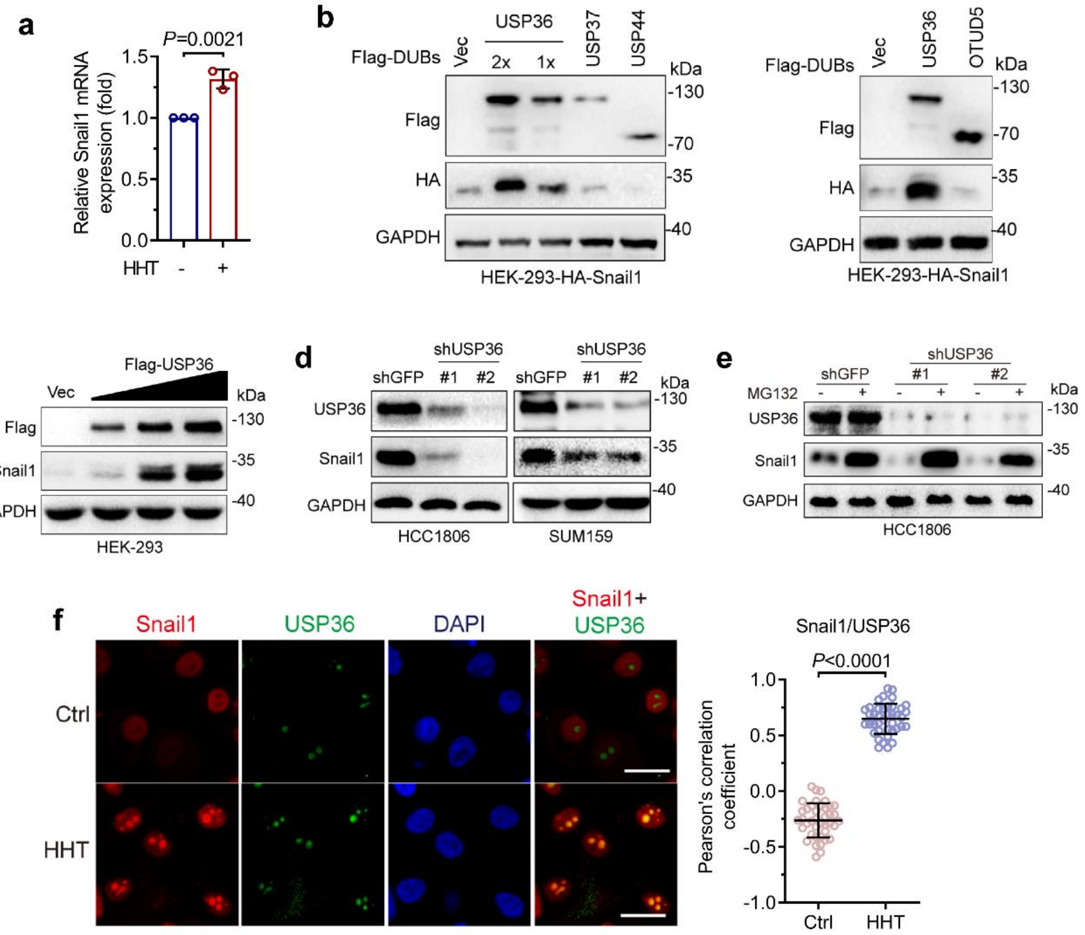

Supplementary Figure S1. USP36 promotes Snail1 protein expression. (a) HCC1806 cells were treated with or without HHT (20 ng/mL) for 24 h. Cells were subjected to qPCR analyses for Snail1 mRNA expression (n=3 biologically independent samples). **(b)** HEK-293 cells stably expressing HA-Snail1 were transiently transfected with a plasmid encoding a deubiquitinase as indicated or vector control (Vec) for 48 h. Cells were subjected to western blot analyses. **(c)** HEK-293 cells were co-transfected with HA-Snail1 and an increased dose of Flag-USP36 for 48 h. Cells were subjected to western blot analyses. **(d)** HCC1806 or SUM159 cells stably expressing shUSP36 (#1 or #2) or shGFP were subjected to western blot analyses. **(e)** HCC1806 cells stably expressing shUSP36 (#1 or #2) were treated with or without proteasome inhibitor MG132 (10 μM) for 6 h. Cells were subjected to western blot analyses. **(f)** HCC1806 cells were treated with or without HHT (20 ng/mL) for 24 h. Cells were subjected to immunofluorescent staining for co-localization of USP36 and Snail1. Quantification of the co-localization between Snail1 and B23 using Pearson's correlation coefficient. Quantification was carried out on 40 cells derived from three independent experiments. Scale bar, 25 μm. These experiments have been repeated for three times with similar results (b-e). Data were presented as mean ± SD and comparisons were performed with unpaired two-tailed Student's t-test (a and f).

Supplementary Figure S2

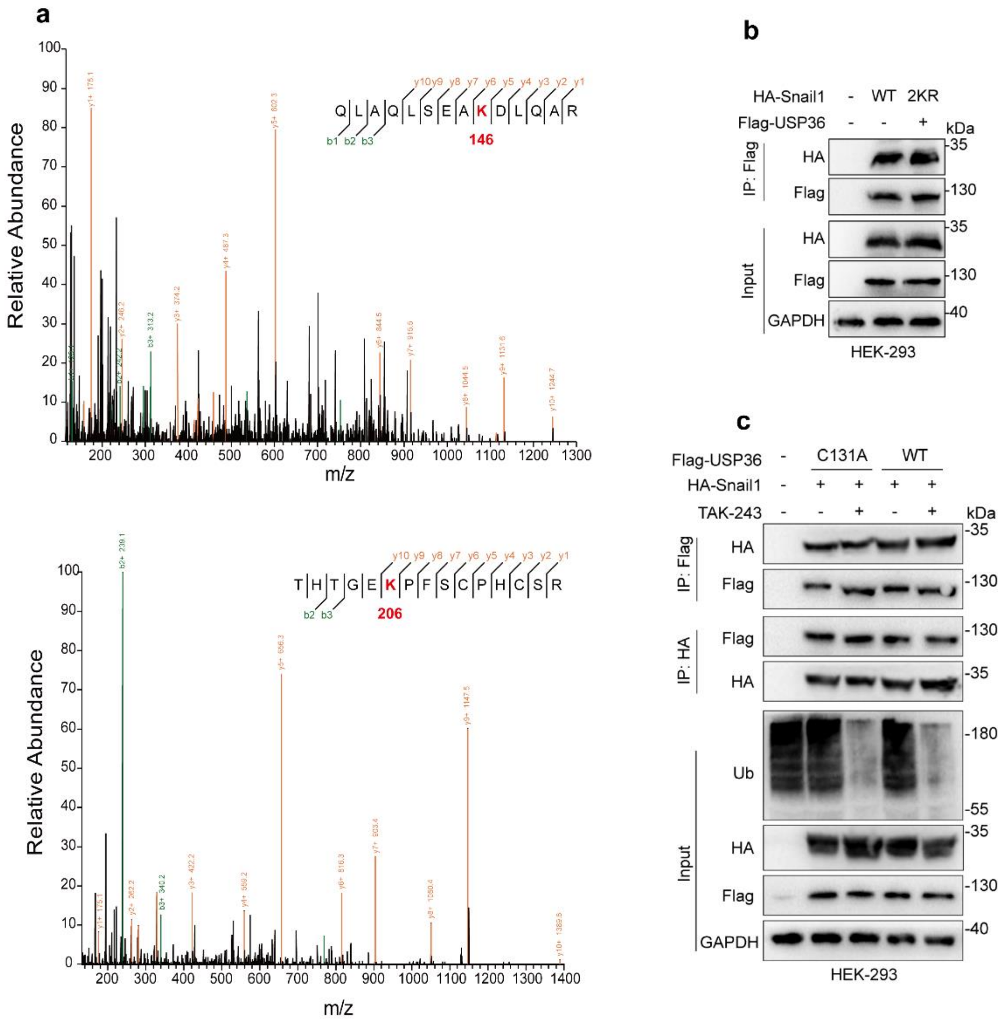

Supplementary Figure S2. K146 and K206 of Snail1 were ubiquitinated and USP36 interacted with Snail1 regardless of the status of its ubiquitination. (a) Representative fragmentation spectrum of the identified Snail1 protein ubiquitination sites. **(b)** HEK-293 cells stably expressing HA-Snail1 (WT or 2KR) were transiently transfected with Flag-USP36 for 48 h. Cells were treated with MG132 (10 μM) for 6 h followed by immunoprecipitation-western blot analyses. **(c)** HEK-293 cells stably expressing HA-Snail1 were transiently transfected with Flag-USP36 (WT or C131A) for 48 h. Cells were treated with or without E1 inhibitor TAK-243 (500 nM) for 8 h prior to MG132 (10 μM) treatment for 6 h followed by immunoprecipitation-western blot analyses. These experiments have been repeated for three times with similar results (b-c).

Supplementary Figure S3

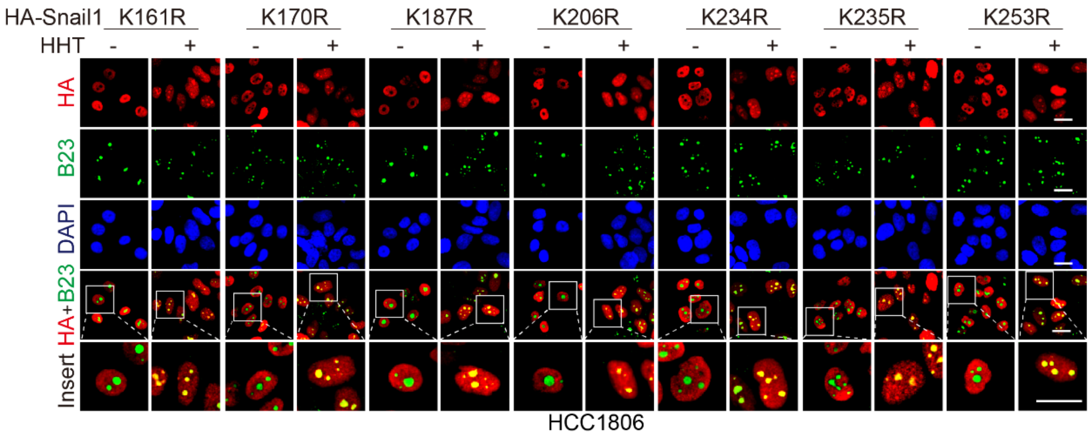

Supplementary Figure S3. HHT promotes Snail1 accumulation in the nucleoli. HCC1806 cells stably expressing HA-Snail1^{K161R}, HA-Snail1^{K170R}, HA-Snail1^{K187R}, HA-Snail1^{K206R}, HA-Snail1^{K234R}, HA-Snail1^{K235R}, or HA-Snail1^{K253R} were treated with or without HHT (20 ng/mL) for 24 h. Cells were subjected to immunofluorescence staining. Notice that all the point mutant proteins could be accumulated in the nucleoli upon HHT treatment. This experiment has been repeated for three times with similar results. Scale bar, 25 µm.

Supplementary Figure S4

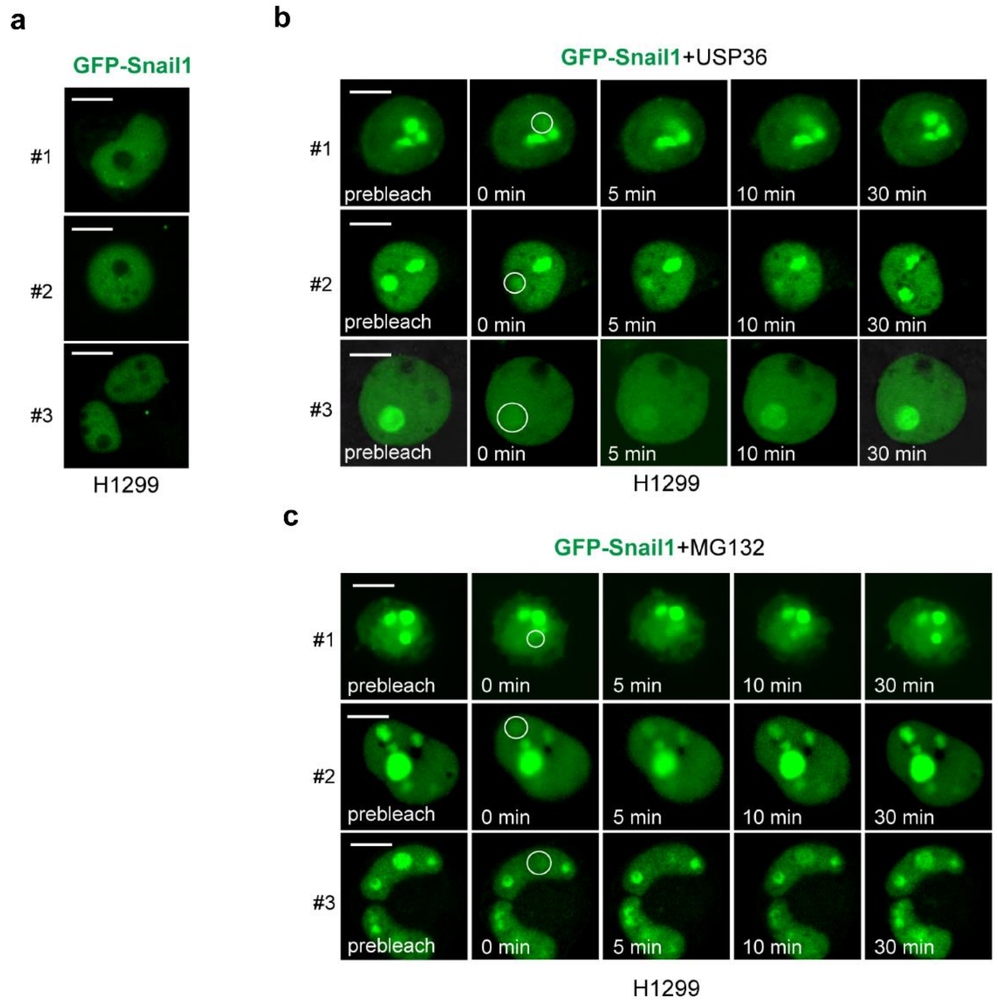

Supplementary Figure S4. Snail1 protein translocates into nucleolus by diffusion in a USP36-independent manner. (a) H1299 cells stably expressing Snail1-GFP were image-captured. **(b)** H1299 cells stably expressing Snail1-GFP were transiently transfected with USP36 for 48 h. Cells were image-captured before and during recovery after the bleaching of a part of the nucleolus (the bleached areas are outlined). **(c)** H1299 cells stably expressing Snail1-GFP were treated with MG132 (10 µM) for 6 h. Cells were image-captured before and during recovery after the bleaching of a part of the nucleolus (the bleached areas are outlined). These experiments have been repeated for three times with similar results (a-c). Scale bar, 25 µm.

Supplementary Figure S5

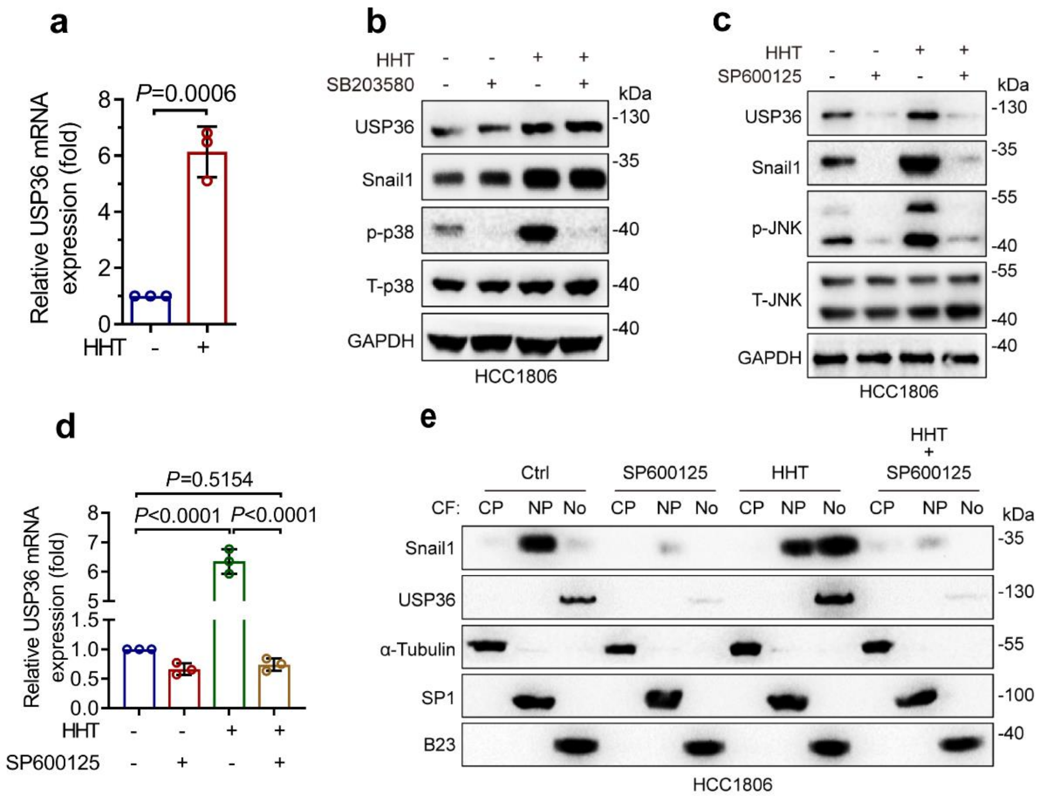

Supplementary Figure S5. Ribotoxic stress promotes USP36 expression via activating the JNK signaling. (a) HCC1806 cells were treated with or without HHT (20 ng/mL and hereafter) for 24 h. Cells were subjected to qPCR analyses for USP36 mRNA expression (n=3 biologically independent samples). **(b)** HCC1806 cells were treated with or without HHT in the presence or absence of a p38 inhibitor SB203580 (10 μM) for 24 h. Cells were subjected to western blot analyses. T-p38: total p38; p-p38: phospho-p38 (Thr180/Tyr182). **(c-d)** HCC1806 cells were treated with or without HHT in the presence or absence of a JNK inhibitor SP600125 (20 μM and hereafter) for 24 h. Cells were subjected to western blot analyses (c) or qPCR analyses (d, n=3 biologically independent samples). T-JNK: total JNK; p-JNK: phospho-JNK (Thr183/Tyr185). **(e)** HCC1806 cells were treated with or without HHT in the presence or absence of SP600125 for 24 h followed by cell fractionation procedure and western blot analyses. CP: Cytoplasm; NP: Nucleoplasm; No: Nucleolus. These experiments have been repeated for three times with similar results (b-c and e). Data were presented as mean ± SD and comparisons were performed with unpaired two-tailed Student's t-test (a) or one-way ANOVA with Tukey's test (d).

Supplementary Figure S6

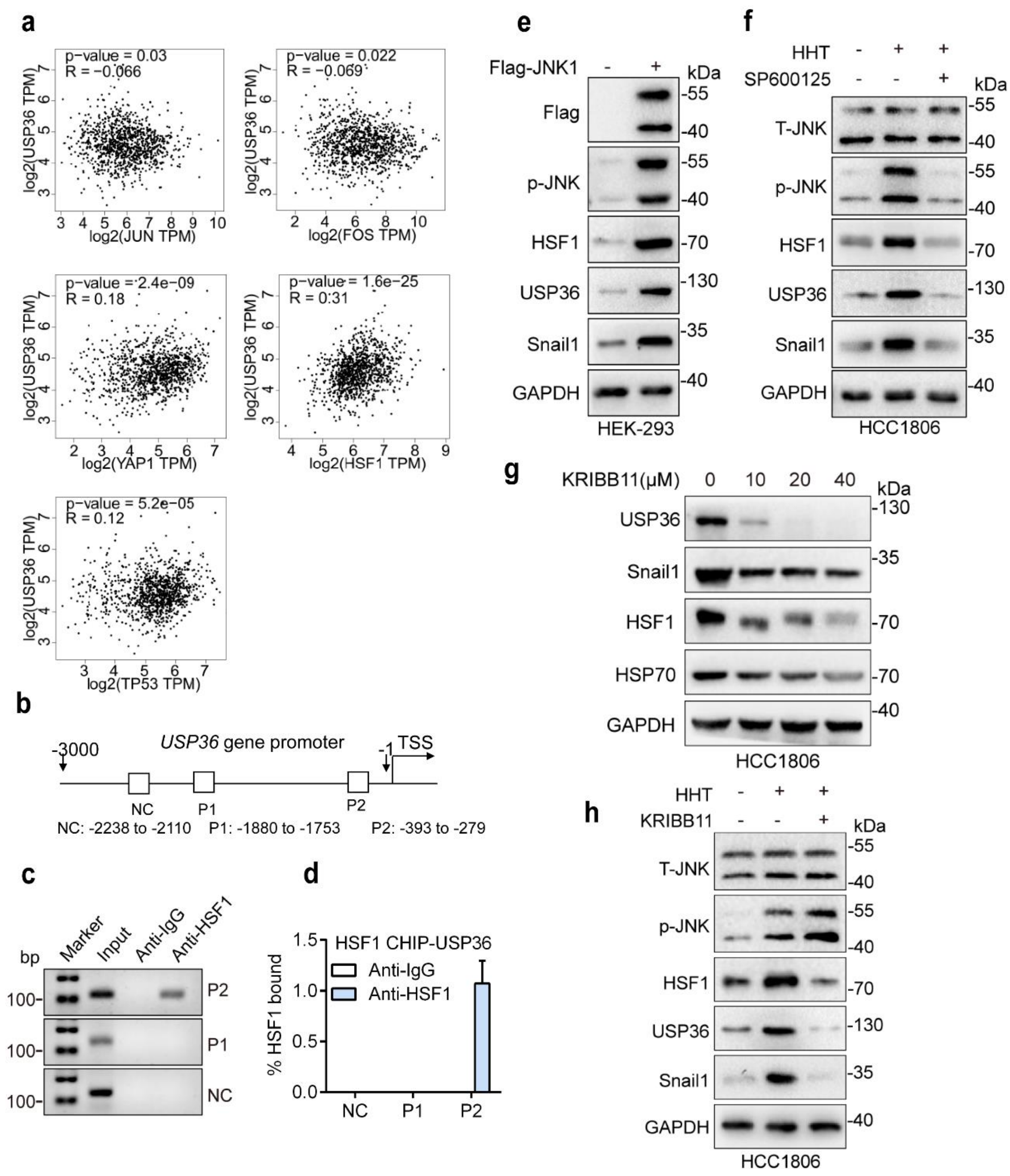

Supplementary Figure S6. Ribotoxic stress activates the JNK-HSF1 signaling to promote USP36 transcription. (a) The GEPIA 2 database (http://gepia2.cancer-pku.cn) was used to analyze the clinical relevance between JUN, FOS, TP53, YAP1, or HSF1, and USP36 in mRNA levels in breast cancers. **(b-d)** A schematic presentation depicts two putative HSF1-binding elements (P1 and P2) on the *USP36* gene promoter (b). Chromatin immunoprecipitation (ChIP) analyses using a specific HSF1 antibody or a control IgG were performed in HCC1806 cells. Specific primers for P1, P2, or negative control (NC) were used. Data derived from reverse transcription-PCR (RT-PCR) (c) or qPCR analyses (d) were shown. TSS: Transcription Start Site. **(e)** HEK-293 cells were transiently transfected with Flag-JNK1 for 48 h. Cells were subjected to western blot analyses. **(f)** HCC1806 cells were treated with or without HHT (20 ng/mL and hereafter) in the presence or absence of SP600125 (10 μM) for 24 h. Cells were subjected to western blot analyses. **(g)** HCC1806 cells were treated with an HSF1 inhibitor KRIBB11 (0, 10, 20, or 40 μM) for 24 h. Cells were subjected to western blot analyses. **(h)** HCC1806 cells were treated with or without HHT in the presence or absence of KRIBB11 (20 μM) for 24 h. These experiments have been repeated for three times with similar results (c-h). Data were presented as mean ± SD (d).

Supplementary Figure S7

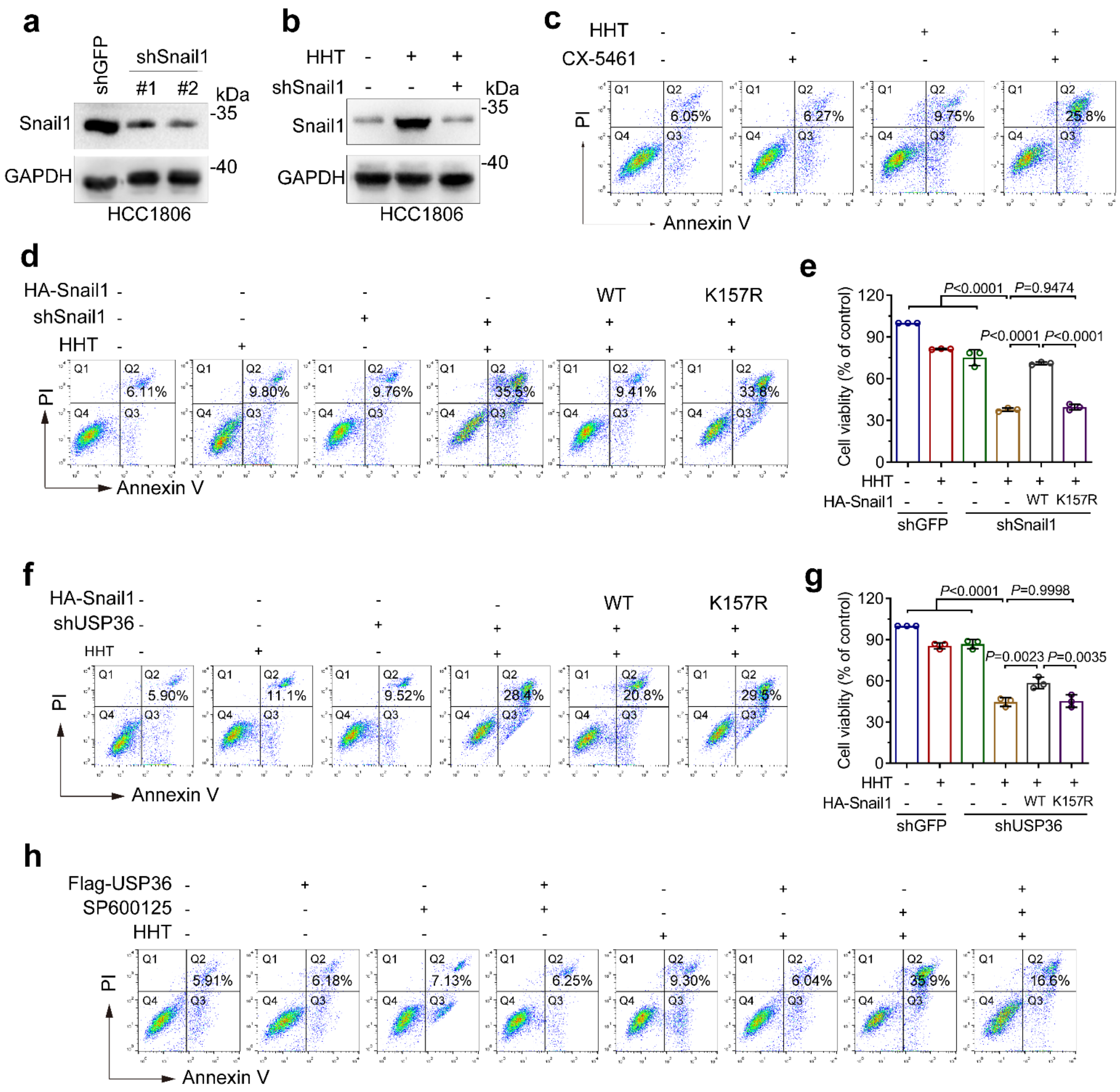

Supplementary Figure S7. Activation of the nucleolar USP36-Snail1 axis promotes cancer cell survival in response to ribotoxic stress. (a) HCC1806 cells stably expressing shGFP or shSnail1 (#1 or #2) were subjected to western blot analyses. **(b)** HCC1806 cells stably expressing shSnail1 were treated with HHT (20 ng/mL and hereafter) for 24h. Cells were subjected to western blot analyses. **(c)** HCC1806 cells were treated with or without HHT in the presence or absence of CX-5461 (200 nM) for 48 h, followed by FACS analyses. **(d)** HCC1806 cells stably expressing shSnail1 were infected with a recombinant lentivirus encoding HA-Snail1^{WT} or HA-Snail1^{K157R}. Cells were treated with or without HHT for 48 h, followed by FACS analyses. **(e)** HCC1806 cells stably expressing shSnail1 were infected with a recombinant lentivirus encoding HA-Snail1^{WT} or HA-Snail1^{K157R}. Cells were treated with or without HHT followed by MTS analyses (n=3 biologically independent samples). **(f)** HCC1806 cells stably expressing HA-Snail1 or HA-Snail1^{K157R} were infected with lentivirus encoding shUSP36. Cells were treated with or without HHT for 48 h. Cells were subjected to FACS analyses. **(g)** HCC1806 cells stably expressing HA-Snail1^{WT} or HA-Snail1^{K157R} were infected with a recombinant lentivirus encoding shUSP36. Cells were treated with or without HHT, followed by MTS (n=3 biologically independent samples). **(h)** HCC1806 cells stably expressing Flag-USP36 were treated with or without HHT in the presence or absence of SP600125 (20 μM) for 48 h. Cells were subjected to FACS analyses. These experiments have been repeated for three times with similar results (a-c, d, f, and h). Data were presented as mean ± SD and comparisons were performed with one-way ANOVA with Tukey's test (e and g). The percentage of apoptotic cells was calculated by Annexin V+/PI+ cells (Q2) (c-d, f, and h).

Supplementary Figure S8

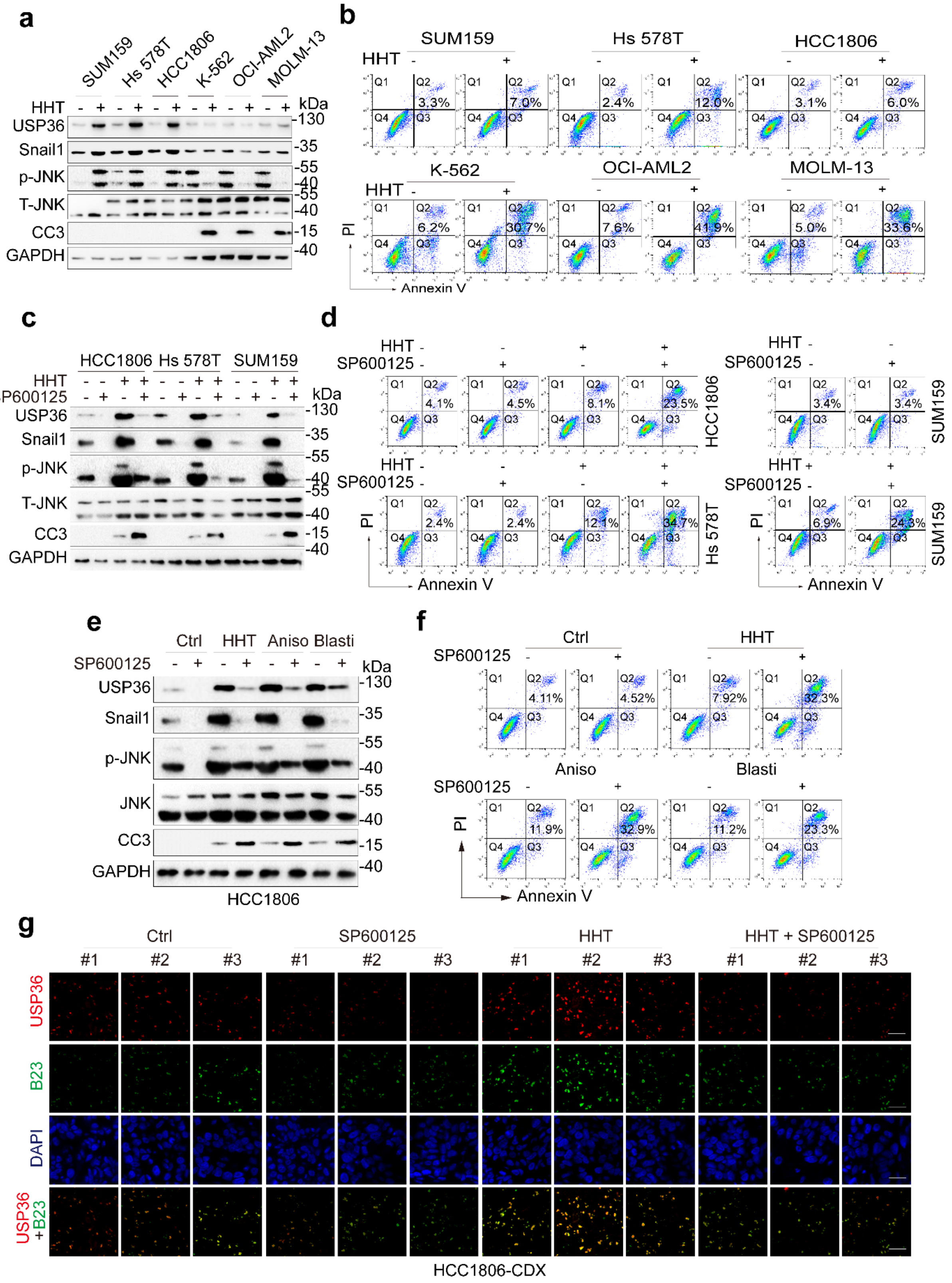

Supplementary Figure S8. Inhibition of the JNK-USP36-Snail1 signaling sensitizes solid tumor cells to HHT. (a-b) Triple-negative breast cancer (SUM159, Hs 578T, and HCC1806) and non-lymphocytic leukemia (K-562, OCI-AML2, and MOLM-13) cells were treated with or without HHT (20 ng/mL and hereafter). Cells were subjected to western blot analyses (a) or FACS analyses (b). **(c-d)** SUM159, Hs 578T, or HCC1806 cells were treated with or without SP600125 (20 μM and hereafter) in the presence or absence of HHT. Cells were subjected to western blot analyses (c) or FACS analyses (d). **(e-f)** HCC1806 cells were treated with HHT, anisomycin (Aniso, 50 ng/mL), or blasticidin (Blasti, 2 μg/mL) in the presence or absence of SP600125. Cells were subjected to western blot analyses (e) or FACS analyses (f). **(g)** Tumor sections derived from Figure 7J were subjected to immunofluorescence staining analyses for USP36 expression. These experiments have been repeated for three times with similar results (a-f). The percentage of apoptotic cells was calculated by Annexin V+/PI+ cells (Q2) (b, d, and f).

Supplementary Figure S9

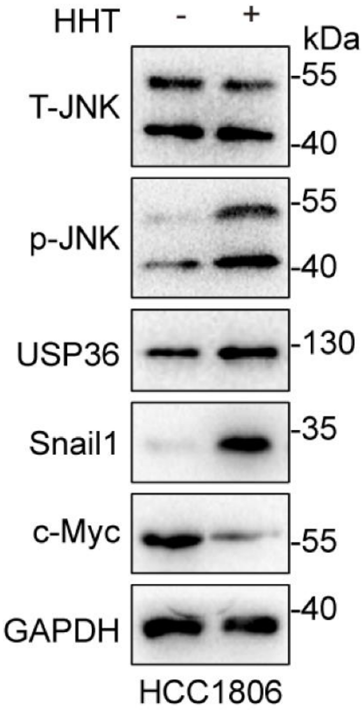

Supplementary Figure S9. HHT inhibits c-Myc expression. HCC1806 cells were treated with or without HHT (20 ng/mL) for 24h. Cells were subjected to western blot analyses. This experiment has been repeated for three times with similar results.

Supplementary Figure S10

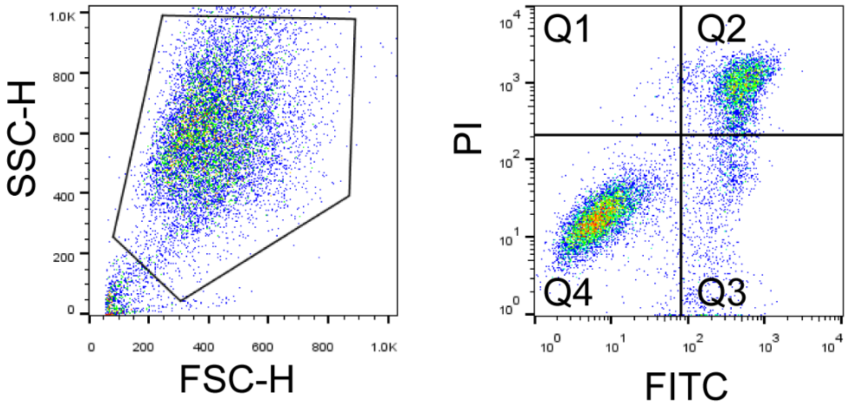

Supplementary Figure S10. The representative FACS gating strategies for analyzing Annexin V-FITC/ PI staining were shown.

Supplementary Table 1. The nucleolar Snail1 positive cells derived from Figure 1B and 1F out of approximately 200 cells were counted. The percentage of positive cells (%) was presented.

Group	Total HCC1806 cells	Nucleolar Snail1 positive HCC1806 cells	Percentage of Nucleolar Snail1 positive HCC1806 cells (%)
Ctrl	197	8	4.06
HHT	197	194	98.48
Anisomycin	201	197	98.01
Puromycin	208	202	97.12
G418	216	211	97.69
Blasticidin	217	213	98.16

Supplementary Table 2. A substantial amount of Snail1 proteins were attributable to the nucleolus.

Cell number	Fluorescence intensity in nucleoli	Fluorescence intensity in nucleus	Nucleolar/Nuclear fluorescence intensity (%)	Mean (%)
#1	21.906	115.648	18.94	18.24
#2	24.92	120.161	20.74	
#3	20.579	116.95	17.60	
#4	15.286	97.532	15.67	

Nucleolar Snail1 positive HCC1806 cells (n=4) derived from Figure 1A were subjected to quantify the fluorescence intensity of nucleolar Snail1 and fluorescence intensity of nuclear Snail1 by Image J. The percentage (%) of fluorescence intensity of nucleolar Snail1/fluorescence intensity of nuclear Snail1 was presented in each of the 4 cells.

Supplementary Table 3. Putative nucleoli DUBs detected by immunofluorescence staining from www.proteinatlas.org/humanproteome/cell/nucleoli.

Gene	Nucleolar localization verified	Nucleolar substrates
USP35	No	Unkown
USP36	Yes[1, 2, 3]	B23, c-Myc, Fibrillarim
USP37	No	Unkown
USP44	No	Unkown
USP46	No	Unkown
OTUD5	No	Unkown
OTUD7A	No	Unkown
MPND	No	Unkown
ATXN3	No	Unkown

Supplementary Table 4. Primers used in this study.

Targets	Sequence	Application
GFP	5′-GAAGCAGCACGACTTCTTC-3′	shRNA
Human Snail1	#1: 5′-CCACTCAGATGTCAAGAAGTA-3′<br>#2: 5′-CCAGGCTCGAAAGGCCTTCAA-3′	shRNA
Human USP36	#1: 5′-GCGGTCAGTCAGGATGCTATT-3′<br>#2: 5′-ACAGAACATCCAACGTCTTAA-3′	shRNA
Human GAPDH	F: 5′-TGGACTCCACGACGTACTCA-3′<br>R: 5′-GAGGGGCCATCCACAGTCTTCT-3′	qPCR
Human USP36	F: 5′-AGCACTTTTCCCCCAGAACTG-3′<br>R: 5′-GGCTCCCAGATCTGCTGCTA-3′	qPCR
Human Snail1	F: 5′-GGCCCTGGCTGCTACAAGGC-3′<br>R: 5′-CTCGAGGGTCAGCGGGGACA-3′	qPCR
Human 47S pre-rRNA	F: 5′-TGTCAGGCGTTCTCGTCTC-3′<br>R: 5′-AGCACGACGTCACCACATC-3′	qPCR
Human E-cadherin	F: 5′-CGAGAGCTACACGTTCACGG-3′<br>R: 5′-GGGTGTCGAGGGAAAAATAGG-3′	qPCR
Human USP36<br>NC	F: 5′-GTCTGCACATCCCAGGTGAGTCTG-3′<br>R: 5′-GCCTTTTCTCATGAGAATTATCAAAGACTTCTCACC-3′	ChIP
Human USP36<br>P1	F: 5′-AAGATGGGGTCTCACGATGTTGC-3′<br>R: 5′-GTCGTCCCAGATGCTTGGGAG-3′	ChIP
Human USP36<br>P2	F: 5′-CCTGTTTTGGAGCAGACATTTGGACC-3′<br>R: 5′-CCCTGCCATGTGCATCTCTTCATC-3′	ChIP
Human 47S pre-rRNA	5’-CGGAGGCCCAACCTCTCCGACGACA<br>GGTCGCCAGAGGACAGCGTGTCAGC-3’	Northern<br>blotting

Supplementary References

1. Endo A, Kitamura N, Komada M. Nucleophosmin/B23 regulates ubiquitin dynamics in nucleoli by recruiting deubiquitylating enzyme USP36. *The Journal of biological chemistry* **284**, 27918-27923 (2009).

2. Sun XX, He X, Yin L, Komada M, Sears RC, Dai MS. The nucleolar ubiquitin-specific protease USP36 deubiquitinates and stabilizes c-Myc. *Proceedings of the National Academy of Sciences of the United States of America* **112**, 3734-3739 (2015).

3. Endo A, *et al.* Nucleolar structure and function are regulated by the deubiquitylating enzyme USP36. *Journal of cell science* **122**, 678-686 (2009).
