# Supplementary material for: USP36 stabilizes nucleolar Snail1 to promote ribosome biogenesis and cancer cell survival upon ribotoxic stress
Source: Nat Commun. 2023 Oct 13;14:6473. doi: 10.1038/s41467-023-42257-8 (PMC10575996; doi:10.1038/s41467-023-42257-8)
Supplement: Supplementary file 2 — Description of Additional Supplementary Files [file 41467_2023_42257_MOESM2_ESM.pdf]

File Name: Supplementary Data 1

Description: Analyses of the USP36 effects on Snail1 protein ubiquitination by mass spectrum.
